# Unveiling diverse coordination-defined electronic structures of reconstructed anatase TiO_2_(001)-(1 × 4) surface

**DOI:** 10.1038/s41467-024-46570-8

**Published:** 2024-03-14

**Authors:** Xiaochuan Ma, Yongliang Shi, Zhengwang Cheng, Xiaofeng Liu, Jianyi Liu, Ziyang Guo, Xuefeng Cui, Xia Sun, Jin Zhao, Shijing Tan, Bing Wang

**Affiliations:** 1 Hefei National Research Center for Physical Sciences at the Microscale and New Cornerstone Science Laboratory, University of Science and Technology of China, Hefei, Anhui 230026 China; 2 Hefei National Laboratory, University of Science and Technology of China, Hefei, Anhui 230088 China; 3 School of Science and Hubei Engineering Technology Research Center of Energy Photoelectric Device and System, Hubei University of Technology, Wuhan, Hubei 430068 China; 4 School of Physics, Hefei University of Technology, Hefei, Auhui 230009 China

**Keywords:** Surfaces, interfaces and thin films, Electronic properties and materials

## Abstract

Transition metal oxides (TMOs) exhibit fascinating physicochemical properties, which originate from the diverse coordination structures between the transition metal and oxygen atoms. Accurate determination of such structure-property relationships of TMOs requires to correlate structural and electronic properties by capturing the global parameters with high resolution in energy, real, and momentum spaces, but it is still challenging. Herein, we report the determination of characteristic electronic structures from diverse coordination environments on the prototypical anatase-TiO_2_(001) with (1 × 4) reconstruction, using high-resolution angle-resolved photoemission spectroscopy and scanning tunneling microscopy/atomic force microscopy, in combination with density functional theory calculation. We unveil that the shifted positions of O 2*s* and 2*p* levels and the gap-state Ti 3*p* levels can sensitively characterize the O and Ti coordination environments in the (1 × 4) reconstructed surface, which show distinguishable features from those in bulk. Our findings provide a paradigm to interrogate the intricate reconstruction-relevant properties in many other TMO surfaces.

## Abstract (web summary)

By measuring in energy, momentum and real space, the authors unveil diverse coordination environments and electronic structures on the reconstructed anatase TiO_2_(001), giving insights into its structure-property relationship with atomic precision.

## Introduction

Transition metal oxides (TMOs) are one of the most important materials due to their versatile physical and chemical properties, such as magnetism^1,2^, ferroelectricity^3,4^, superconductivity^5,6^, and inspire various applications including (photo)catalysis^7,8^. Tremendous research interest has been attracted to interrogate the complex structure-property relationships at the atomic scale, concerning how the coordination between the metal cation and oxygen anion can contribute to the electronic structures^9,10^. The local coordination environments are determined by the coordination number, bond order, length, and angle. However, the coordination environments suddenly change when the bulk structure terminates at a surface. Reconstructions usually take place at the TMOs’ surfaces to minimize the surface energy, accompanied by the rearrangement of electronic structures^11^. The surface region can usually confine 2D/3D electron gas, providing a playground for correlated electron-electron, electron-boson couplings, and spin-charge interconversion^12,13^. Furthermore, lower coordination could appear at the surface defects and step edges, which are recognized as the active center for catalytic reactions^14,15^. These fascinating phenomena ranging from strong correlation to surface catalysis require deep and comprehensive insights into the coordination environments and the corresponding electronic states.

The precise measurement of the coordination environment and electronic property relies on multiply high resolutions in energy, real and momentum spaces. Surface science characterization techniques have been widely used to measure the geometric and electronic structures of TMOs from different aspects, but each technique has its limitations. In real and momentum spaces, low-energy electron diffraction (LEED) and surface X-ray diffraction (SXRD) can characterize the surface structures^16,17^. Transmission electron microscopy (TEM) can image the cross-sectional atomic arrangements in bulk^18^. Non-contact atomic force microscopy (NC-AFM) with a qPlus sensor^19^ has achieved single-bond resolution^20–22^. Scanning tunneling microscopy (STM) has provided a powerful tool in detecting both the surface structures and local density of states^23,24^. However, STM is mainly sensitive to the electronic states in the vicinity of Fermi level (*E*_F_) at the near-surface region. The broadly distributed core level and valence band (VB) have usually been examined by X-ray and UV photoemission spectroscopies (XPS/UPS), respectively^25,26^. However, the lack of momentum resolution makes it difficult to identify the band dispersions and thus hard to assign the bands directly. Yet, angle-resolved photoemission spectroscopy (ARPES) has rarely been used to measure the deep valence band electronic structures of TMOs^27,28^.

Our study reports that the joint STM-AFM-XPS-UPS-ARPES, in combination with density functional theory (DFT), can provide a comprehensive determination of characteristic electronic structures from diverse coordination environments in the (1 × 4) reconstructed surface of anatase-TiO_2_(001). By analyzing the measured O 2*p* VB, O 2*s* semi-core level, and Ti 3*d* gap state (GS) and comparing with the calculated electronic structures on the basis of various structural models, the surface electronic states are distinguished from the bulk ones, and able to be correlated to the contributions from the different coordination environments. Such an approach by capturing high-resolution global parameters could be widely applied to other intricated TMO materials.

## Results

### Real-space imaging the surface structures of anatase-TiO_2_(001)-(1 × 4) using STM and AFM

**Fig. 1:**
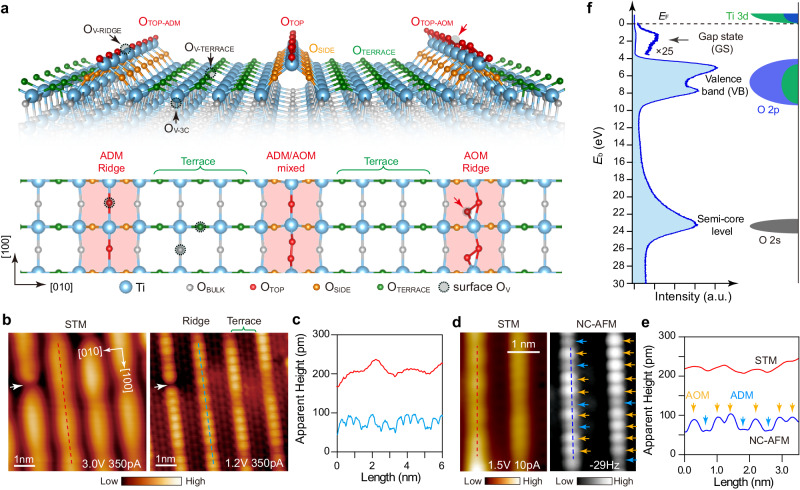
Diverse coordination environments in the (1 × 4) reconstructed surface of anatase-TiO_2_(001). **a** Structural model of the (1 × 4) reconstructed surface. Upper panel: side view, lower panel: top view. The ball-and-stick model sketches three kinds of ridges. Left: the ADM ridge (O_TOP-ADM_), right: the AOM ridge (O_TOP-AOM_), middle: the mixed ADM and AOM ridge with a ratio of 1:1. The O_TOP_, O_SIDE_, O_TERRACE_, and O_BULK_ atoms are correspondingly colored in upper and lower panels. An O_TOP-AOM_ becomes an O_TOP-ADM_ when it is missing an O atom marked by the red arrows. The surface O_V_ defects at terrace (O_V-TERRACE_ and O_V-3C_) and ridge (O_V-RIDGE_) sites are marked by the black arrows. **b** The empty-state STM images of high bias (3.0 V, 350 pA) and high-resolution (1.2 V, 350 pA) with the same area, measured at 80 K. High-resolution STM image exhibits clear terrace and ridge structures. The white arrow indicates an intrinsic dark point defect^32^. **c** Line profiles extracted from the corresponding colored lines in **b**.The red curve from high bias STM image shows the nonuniform electronic distributions, and the cyan one from the high-resolved image presents two different heights. **d** A set of in-situ empty-state STM (1.5 V, 10 pA) and the NC-AFM images at a frequency shift of −29 Hz within the same area, measured at 5 K, using a W tip. **e** Line profiles extracted from the corresponding colored lines in **d**. The red curve from STM image shows the nonuniform electronic distributions, and the blue one from AFM presents two distinct contrasts, labeled as ADM (blue arrow) and AOM (yellow arrow), according to the relative height in their models. **f** Left panel: a representative XPS spectrum in the energy range 0–30 eV measured at excitation with light energy of *hυ* = 200 eV, sample kept at 20 K, showing the main features of GSs, VBs, and semi-core levels. Right panel: schematic diagram of the main contributions from Ti *3d*, O *2p*, and O *2s*.

The bulk anatase-TiO_2_ consists of 6-fold coordinated Ti atoms and 3-fold coordinated O atoms (O_BULK_). Its terminating (001) surface involves the reduced coordination of 5-fold Ti atoms and the bridging 2-fold O atoms, endowing high surface energy for the well-studied catalytic applications^29^. However, surface science studies employ ultrahigh vacuum annealing that causes the release of (1 × 1) surface energy, leading to the (1 × 4) reconstruction at anatase-TiO_2_(001) surface^30^. The (1 × 4) superlattice consists of alternated terraces (O_TERRACE_) and hunching-up ridges, where the ridge involves different O rows at the side (O_SIDE_) and the top (O_TOP_) (Fig. 1a). There are two models, namely “ADM”^31^ and “AOM”^32^, that describe the possible structures of O_TOP_. The main difference is that the ADM ridge involves single O_TOP_ row with 2-fold coordination (O_TOP-ADM_)^31,33,34^, while the AOM ridge involves double O_TOP_ rows with 3-fold coordination (O_TOP-AOM_)^32,35^. The theory also predicts the possible coexistence of ADM and AOM ridge sites^36,37^ (sketched in Fig. 1a). In experiments, the STM images give some signature for their coexistence, for example, the contrast at ridge is quite nonuniform (Fig. 1b, c). However, the STM resolution is not enough to distinguish the ADM and AOM directly^32^, remaining a problem to be clarified.

We perform in situ STM and AFM experiments to characterize the structures at a high resolution. The empty-state STM image mainly integrates the Ti electronic state on the ridge and terrace (Fig. 1d, left panel), while the AFM image (given in inverted contrast because of attraction force regime in imaging) mainly reflect the contrasts of O atoms along the ridges (Fig. 1d, right panel). The two groups of O atoms can be distinguished from their relative contrasts, as labeled by ADM and AOM according to the relative heights in their structural models^32,38^ (Fig. 1e). Both the STM and the AFM images indicate the coexistence of the ADM and AOM structures on the ridge.

Actually, oxygen vacancy (O_V_) defects can appear on the surface at either terrace or ridge sites, which act as the reactive centers and introduce metallic states and GSs^39,40^. In Fig. 1a, these coordination environments for O atoms in the anatase-TiO_2_(001)-(1 × 4) surface are labeled by O_BULK_, O_TERRACE_, O_SIDE_, O_TOP-ADM_, O_TOP-AOM_, O_V_ at ridge (O_V-RIDGE_), and at terrace (O_V-TERRACE_ and O_V-3C_). They together contribute to the complex electronic states, as shown by the measured XPS spectrum (Fig. 1f), which presents a weak Ti *3d* GS between the Fermi level (*E*_F_) and VB maximum (VBM), the intense VB mainly consisting of O *2p* levels, and a broad peak consisting of O *2s* semi-core levels. We now attempt to correlate these electronic states with their coordination environments.

### Distinguishing the surface bands from momentum-resolved O *2p* spectra

**Fig. 2:**
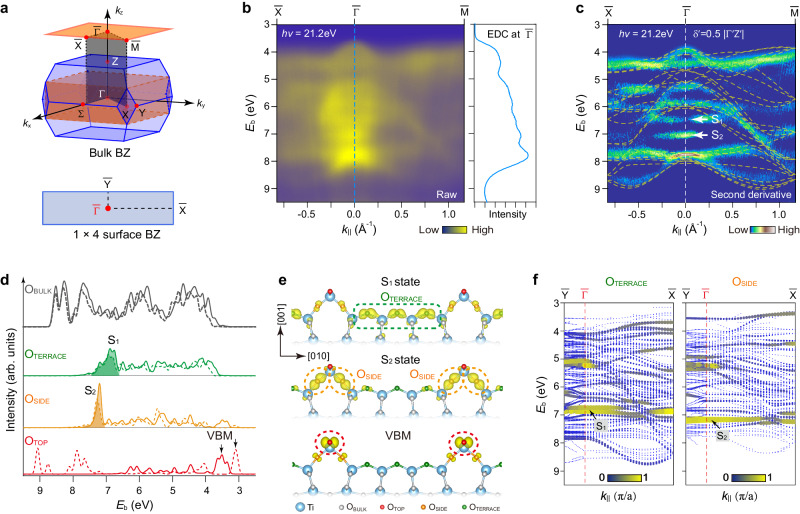
Distinguish the bulk and surface VBs from momentum-resolved O *2p* spectra. **a** Upper panel: The bulk BZ and the projected surface BZ. Blue shade indicates the bulk *bct* structure of primitive cell. Orange shade indicates the bulk *st* structure of unit cell. The top orange shade is the projected surface 2D structure. Lower panel: surface (1 × 4) reconstructed BZ. **b** The raw ARPES cut excited by *hυ* = 21.2 eV along the high-symmetry axes and the corresponding EDC at $\bar{\Gamma }$ point. **c** The second derivative cut from **b**, overlaid with the calculated bulk VBs at the corresponding *k*_*z*_ (${{{{{\rm{\delta }}}}}}^{\prime} \sim 0.5$) (yellow dashed lines, see details in Supplementary Fig. 1). The calculated VBs have been shifted to maximize the overlap with the experimental results. White arrows: surface levels of S_1_, S_2_ bands. **d** The calculated pDOSs of O_BLUK_, O_TERRACE_, O_SIDE_, O_TOP-ADM_ and O_TOP-AOM_, based on the ADM (solid lines) and AOM (dotted lines) models. **e** The extracted electronic states of S_1_, S_2_ and VBM bands of the ADM model at $\bar{\Gamma }$ point, exhibiting localized spatial distributions at the O_TERRACE_, O_SIDE_, and O_TOP-ADM_ atoms, respectively. **f** Calculated VB dispersion according to the weightings of surface O_TERRACE_ and O_SIDE_ sites, respectively. The O_TERRACE_ bands involve the main feature of S_1_ and the O_SIDE_ involve the main feature of S_2_, respectively.

The anatase-TiO_2_ has a body-centered-tetragonal (*bct*) structure with the space group *I*4_1_/*amd*^41^. The bulk *bct* Brillouin zone (BZ) of primitive cell (shaded in light blue) is depicted in Fig. 2a. A simple tetragonal (*st*) BZ of unit cell (shaded in orange) is usually applied to examine the band structures along the high symmetric axes^42^, which is projected to a surface BZ with (1 × 4) reconstruction along the high symmetric points of $\bar{\Gamma }$, $\bar{{{{{{\rm{M}}}}}}}$ and $\bar{{{{{{\rm{X}}}}}}}$. As known for most TMOs, the VB of anatase-TiO_2_(001) involves the main contribution from the O *2p* states and a minor contribution from the Ti *3d* states^43^, as shown in the spectrum in Fig. 1f. However, such momentum-integrated spectrum cannot clearly resolve the exact bands. We present the 2D energy-momentum [*E*(*k*_*||*_)] resolved VB map along the high-symmetry $\bar{\Gamma }-\bar{{{{{{\rm{X}}}}}}}$ and $\bar{\Gamma }-\bar{{{{{{\rm{M}}}}}}}$ directions, with the binding energy (*E*_b_) mainly distributing from ~3.0 to 9.0 eV below *E*_F_ and the energy distribution curve (EDC) at $\bar{\Gamma }$ point (Fig. 2b). Using its second derivative map^44^, the contrasts of the band structure in the ARPES map can be highly improved (Fig. 2c). To assign the observed bands, the bulk electronic bands have been calculated by using both the primitive cell and the unit cell^41,42^, as well as different *k*_z_ (see calculation details in Supplementary Fig. 1). The calculated bulk VBs can overall reproduce the experimental results, except for the two at *E*_b_ ~ 6.5 eV and 7.1 eV, as labeled by S_1_ and S_2_, respectively (Fig. 2c).

We turn to consider the S_1_ and S_2_ as the VBs contributed by surface O atoms. To verify their surface nature, we carried out the experiment by depositing potassium (K) atoms to the surface because the alkali atoms can strongly modify the surface levels but have little influence on the bulk bands. It is clear that the S_1_ and S_2_ can be quenched by K atoms adsorption and recovered after K desorption, similar to behaviors of the H_2_ adsorption on Ir surface^45^, while the bulk bands remain unchanged (Supplementary Fig. 2). Different from the bulk VBs with broadly distributed *E*(*k*_*||*_) dispersions, the S_1_ and S_2_ are nearly flat near $\bar{\Gamma }$ point. Such unique features imply the S_1_ and S_2_ may be contributed by certain specific O sites in the surface. We calculated the surface electronic structures using the slab models by including the geometries of ADM and AOM models, respectively. The projected density of states (pDOSs) of each O atoms of O_TERRACE_, O_SIDE_, O_TOP-ADM_ and O_TOP-AOM_ are extracted to distinguish their contributions (Fig. 2d). For O_TERRACE_ and O_SIDE_ atoms, it is found that both ADM (solid lines) and AOM (dotted lines) models give approximate electronic structures within a broadly hybridized energy range of *E*_b_ ~ 3–8 eV. But, the O_TERRACE_ atoms dominate at *E*_b_ ~ 6.8 eV (green shaded) and the O_SIDE_ atoms dominate at *E*_b_ ~ 7.2 eV (orange shaded) with well-defined spatial distributions (Fig. 2e), which could be readily assigned to the observed S_1_ and S_2_ bands, respectively. Furthermore, we plot the band dispersion of each O atoms calculated using the ADM model (Supplementary Fig. 3). The weightings of surface O atoms show localized features relative to the that of bulk O atoms. The weightings of S_1_ and S_2_ indicate near flat character in the vicinity of $\bar{\Gamma }$ point (Fig. 2f), in good agreement with those observed in the experimental *E*(*k*_*||*_) map. For O_TOP_ atoms, it is known that they mainly contribute to the VBM^46,47^ with localized features (Fig. 2e). The calculated pDOS of O_TOP-ADM_ and O_TOP-AOM_ with ADM and AOM models, respectively, dominate at VBM, but with slight energy difference (Fig. 2d). From the O *2p* spectra, it is still difficult to distinguish O_TOP-ADM_ and O_TOP-AOM_, we thus search for more evidence from the O *2s* spectra in the following sections.

### Recognizing the surface semi-core levels from O *2s* spectra

**Fig. 3:**
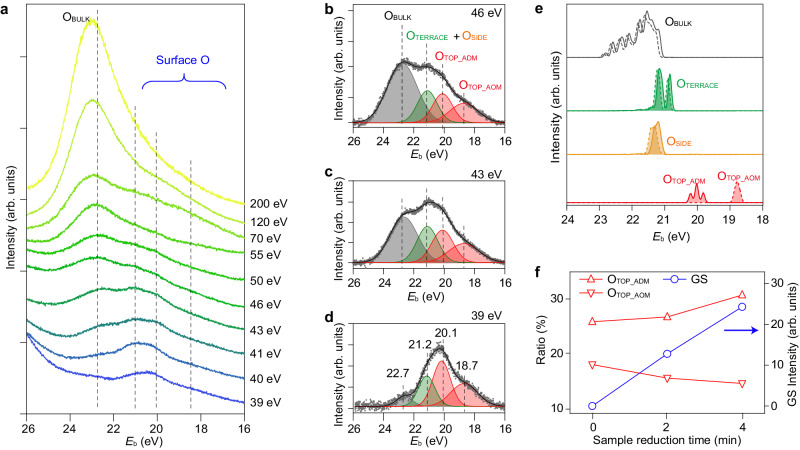
Recognizing the surface core levels from O *2s* spectra. **a** Evolution of O *2s* semi-core level spectra with increasing excitation photon energy from 39 eV to 200 eV. **b**–**d** Peak fitting of O *2s* spectra with BG subtracted for 39, 43, and 46 eV excitation, respectively. Four peaks at 22.7, 21.2, 20.1, and 18.7 eV can be extracted with relative intensity changes. **e** The calculated O *2s* semi-core level pDOSs of each O atom. **f** Plot of the O_TOP-ADM_ (red triangle) and O_TOP-AOM_ (red inverted triangle) contributed peak area ratio in the left axis and the corresponding Ti *3d* GS intensity (blue circle) in the right axis as a function of sample reduction. The as-grown sample was gradually reduced by increasing Ar^+^ sputtering (each cycle 2 min) and annealing at 650 °C.

The O *1s* XPS spectrum of anatase-TiO_2_(001)-(1 × 4) exhibits only a single peak, as widely observed in the crystalline TiO_2_ surfaces^48,49^. By contrast, even under excitation by a high photon energy (610 eV), the O *2s* spectrum of anatase-TiO_2_(001)-(1 × 4) presents a tail spreading to a low *E*_b_ region, where the tail could be from the contributions of the surface O atoms (Supplementary Fig. 4). Distinctly different features can be observed in comparison with the results from a simpler rutile-TiO_2_(110)-(1 × 1) surface at excitations using lower photon energies, which can be more sensitive to the surface states (Supplementary Fig. 5a, b). By tuning the excitation photon energies (*hυ*) from 39 eV to 200 eV, the evolution of these peaks can be more clearly shown (Fig. 3a). According to the universal curve for the electron inelastic mean free path (IMFP) as a function of the kinetic energy^50^, it can be expected that the spectra excited with *hυ* > 100 eV could mainly detect the bulk information, while those excited with *hυ* ~ 30–100 eV are sensitive to surface region. The spectra excited with *hυ* = 120 and 200 eV show a strong peak at *E*_b_ ~ 22.7 eV, which could be assigned to the contributions of O_BULK_ atoms (Fig. 3a), similar to the results from the rutile TiO_2_(110) surface^51^. As the *hυ* reduced to below 100 eV, the intensity of the peak at *E*_b_ ~ 22.7 eV decreased dramatically, and meanwhile, several new peaks at lower *E*_b_ arise. Three selected spectra with *hυ* = 39, 43 and 46 eV excitation are presented in Fig. 3b–d, which can be decomposed into four peaks by the best XPS fitting (Supplementary Fig. 6). Except for the peak at *E*_*b*_ ~ 22.7 eV that is contributed by O_BULK_ atoms (gray shaded), the three peaks at *E*_b_ ~ 21.2, 20.1, and 18.7 eV could be contributed by surface O atoms, as a result of the chemical shifts^52^ of the surface semi-core levels. It is noted the intensity of these distinguishable peaks are changing dramatically: for the one at *E*_b_ ~ 22.7 eV of O_BULK_ atoms, the enhanced intensity could be reasonably attributed to the increased IMFP with larger *hυ*; for the other three peaks of surface O atoms, we need to consider some other possible factors, like the photoelectron diffraction and the resonant photoemission processes. We can exclude the effect of the photoelectron diffraction due to the possible diffraction-caused enhanced angle-dependent intensity variations (Supplementary Fig. 7). The angle distribution curves (ADCs) at each photon energy give overall Gaussian-like shape for the integrated intensity distributions against *θ*, obviously no diffraction-enhanced intensity with ±15°. The EDCs show nearly the same feature at each excitation photon energy, showing the angle-independent O *2s* spectra. These analyses can exclude the possibility of the diffraction effect by the reconstructed surface structure. While, by measuring the spectra using the tunable excitation phonon energy in the range of *hυ* ~ 39–55 eV, the resonant photoemission processes were observed to occur at around *hυ* ~ 43–46 eV (Supplementary Fig. 5c), which could be assigned to the Ti 3*p* → 3*d* optical transition at anatase-TiO_2_(001) surface^53,54^. Such resonant photoemission processes could enhance the photoemission intensities of the surface semi-core levels at certain excitation photon energy, and make the peaks more distinguishable in the O *2s* spectra. Nevertheless, this effect does not contribute any additional peak and much easily be recognized according to the analysis of our results.

To understand these O *2s* semi-core level peaks, we analyzed the calculated pDOS in Fig. 3e. The pDOS of O_BULK_ has a broad spectral weight in the range *E*_b_ ~ 21–23 eV, which corresponds well to the peak of *E*_b_ ~ 22.7 eV in the experiment. The pDOS of O_SIDE_ and O_TERRACE_ sites overlap in the energy range of *E*_b_ ~ 20.8–21.4 eV (Fig. 3e). These two together could be probably correlated to the peak of *E*_b_ ~ 21.2 eV in the experiment. More interestingly, the pDOS of O_TOP-ADM_ and O_TOP-AOM_ atoms are clearly separated at *E*_b_ ~ 20.1 eV and 18.7 eV, respectively, which are in excellent agreement with the two lower energy peaks in the experiment (red shaded in Fig. 3b–d). Based on the above assignments, the existence of the two peaks at *E*_b_ ~ 20.1 eV and 18.7 eV implies the coexistence of ADM and AOM ridge structures at the anatase-TiO_2_(001)-(1×4) surface, in line with the two distinct contrast of ADM and AOM in the AFM measurements (Fig. 1d).

The quantitative analysis of the mixed ADM and AOM ratio is difficult, because the absolute areas of the two peaks are sensitive to the excitation *hυ* and rely on how the background signal are subtracted. Qualitatively, we investigated the change of ADM and AOM ratio at the excitation *hυ* = 40.8 eV using slightly reduced surface through treatments of sputtering and annealing cycles (Supplementary Fig. 8). The reduction procedure is monitored by the increase of Ti *3d* GS intensities (blue circle) in Fig. 3f. It can be seen that the O_TOP-ADM_ component (red triangle) is increasing while the O_TOP-AOM_ component (red inverted triangle) is decreasing during the sample reduction procedure, which strongly supports our assignments. To further examine the stability of mixed ADM-AOM structure of anatase TiO_2_(001)-(1×4), we calculate the surface energy correlation with the oxygen chemical potential and the phase diagram (Supplementary Fig. 9), in consistent with the similar phase diagrams for AOM and ADM in previous stusies^47,55,56^. Our study provides additional information for the mixed ADM-AOM structures with tunable ratios. The results indicate that the mixed ADM-AOM structure could be a stable phase during sample growth under a relatively low temperature and pressure conditions.

### Identifying the O defect states from Ti *3p* and *3d* spectra

**Fig. 4:**
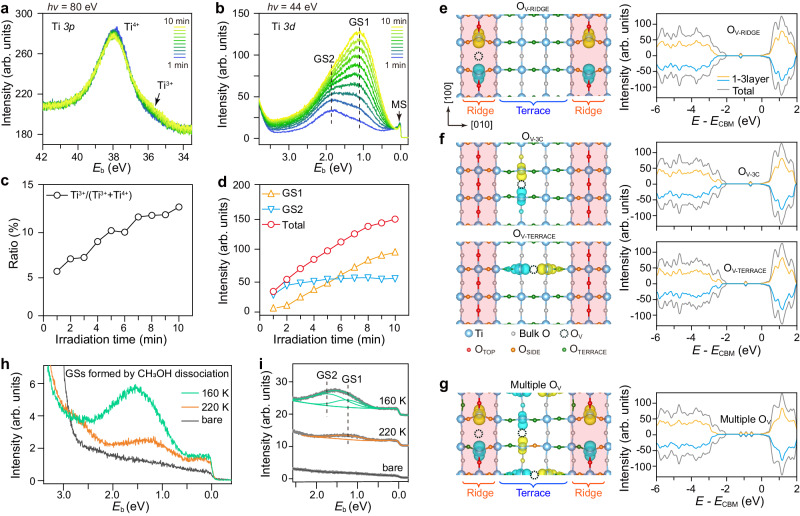
Recognition of different O_V_ defects induced Ti *3d* GSs. **a**, **b** Ti *3p* core level spectra obtained by *hυ* = 80 eV and the in-gap UPS spectra excited by *hυ* = 44 eV (synchrotron radiation light), respectively. **c**, **d** Evolution of the Ti^3+^/(Ti^3+^+Ti^4+^) ratio, and the intensities of GS1, GS2 and total of (GS1 + GS2), as a function of light irradiation time. **e**–**g** Calculated charge density contours of excess electrons and DOSs of total (gray) and 1–3 layer (yellow: spin up, blue: spin down) with an O_V-RIDGE_ at ridge **e**, an O_V-3C_ or O_V-TERRACE_ at terrace **f** and multiple Ov defects at ridge and terrace **g**, respectively. **h**, **i** The in-gap UPS spectra obtained after 30 min UV irradiation (He lamp, 21.2 eV) with CH_3_OH adsorption at 160 and 220 K, respectively. In **i**, the UPS spectra are fitted after background subtraction with one peak at 1.20 eV for the spectrum at 220 K, and double peaks at 1.20 and 1.75 eV for the one at 160 K. A spectrum measured at the bare surface is used for reference (gray).

The O *2p* and 2*s* spectra above have successfully identified the different coordination environments of O atom at the reconstructed anatase-TiO_2_(001)-(1 × 4) surface. In addition, the O_V_ defects commonly exist on oxides’ surface causing excess electron doping at the surface. The electron doping could introduce two features: (i) a delocalized electron gas at near-surface region confined by the band bending potential, which forms a metallic state (MS) just below *E*_*F*_ (refs. ^39,40^); (ii) localized electron denotation to nearby Ti atoms, which reduces Ti^4+^ to Ti^3+^ and forms Ti *3d* GSs^32,39,40^. The O_V_ defects could be produced by either sputtering and annealing in vacuum^32^, or light irradiation with a high flux radiation beam^39^. We here present the evolution of Ti *3p* and *3d* spectra with O_V_ formation under light irradiation. By increasing the light irradiation time, the intensity of Ti^4+^ state gradually decreases while the intensity of Ti^3+^ state arises in the Ti *3p* spectra (Fig. 4a). Meanwhile, both MS and GSs arise in the Ti *3d* spectra (Fig. 4b). The intensities of GSs increase gradually, while that of MS looks rapidly saturating at *E*_*F*_. This is because the MS is formed due to the lowered Ti 3*d* band below *E*_*F*_, which has a saturated electron density that leads to the saturated intensity near the *E*_*F*_ (ref. ^39^). Two distinct GSs appear at *E*_b_ ~ 1.1 and 1.8 eV in the Ti *3d* spectra, as denoted by GS1 and GS2, respectively (Fig. 4b). Different from the rutile-TiO_2_(110)−1 × 1 surface, where the O_V_ defects can only produce one GS at *E*_b_ ~ 0.9 eV^57^, the emergence of two GSs on anatase-TiO_2_(001)-(1 × 4) surface is unique, which had been observed in previous studies^39,40^ but have not been understood yet. Bigi et al. found the adsorption of O_2_ at defective anatase-TiO_2_(001)-(1 × 4) surface can quench both the GS1 and GS2, and thus demonstrate their origin should be from surface defects rather than from bulk defects^40^. We extract the intensities of the two GSs separately from the best fitting (Supplementary Fig. 10). It is found that although the intensities of Ti^3+^ state (Fig. 4c) and the total GSs (Fig. 4d) are increasing meanwhile as a function of light irradiation time, the intensities of GS1 and GS2 are changing asynchronously (Fig. 4d), implying the GS1 and GS2 must have different origins from surface defects. In our previous experiments, we have detected the point defect induced GS at *E*_b_ ~ 0.9 eV at ridge sites by scanning tunneling spectroscopy^32^, and the hydroxyl groups induced GS at *E*_b_ ~ 1.6 eV at terrace sites by UPS^49^. Considering the approximated energies, we assign the observed GS1 at *E*_b_ ~ 1.1 eV and GS2 at *E*_b_ ~ 1.8 eV to the electronics states of O_V_ defects at ridge and terrace sites, respectively.

We calculate the configurations and formation energies for possible surface O_V_ defects at ridge and terrace sites using spin-polarized DFT (Supplementary Fig. 11). From the formation energies, it is found that the missing of one O_TOP_ is the most possible O_V_ defect at the ridge (O_V-RIDGE_, Fig. 4e), and the missing of a bridging O atom along [100] direction or along [010] direction could be the O_V_ defect at terrace (O_V-3C_ and O_V-TERRACE_, Fig. 4f). The calculated electronic structures with PBE + U functional (U = 3.9 eV)^40^ show that each O_V_ defect can contribute to a GS with its excess electron redistributing to adjacent Ti atoms (Fig. 4e, f). Further ab initio molecular dynamics (AIMD) simulations indicate the excess electrons are stable at the adjacent Ti atoms (Supplementary Fig. 12). The charge redistribution and the distortion of the lattice in the vicinity imply the formation of small electron polaron, similar as the small polaron of O_V_ at rutile-TiO_2_(110) surface^58^. But, from the pDOS with either antiferromagnetic^59^ or ferromagnetic states (Supplementary Fig. 13), the energies of different O_V_ defects are not separated clearly, making the assignment difficult. Such energy inaccuracy is possibly because of the self-interaction error in DFT, which usually leads to an underestimated bandgap of TiO_2_. In particular, when multiple O_V_ defects are considered (Supplementary Fig. 14), the GSs show very close energies within the underestimated bandgap of 2.5 eV (Fig. 4g).

It is difficult to create O_V_ defects at either ridge or terrace in a controllable manner for verifying the assignment of GS1 and GS2. Alternatively, we design an experiment to create hydroxyl groups at ridge and terrace sites from methanol (CH_3_OH) dissociation, by considering the hydroxyl groups can provide surface states similar to those of the O_V_ defects owing to the induced excess electrons that contribute to the GSs. Methanol has similar adsorption behavior as water^49^, that the molecular CH_3_OH only adsorbs at ridge sites at *T* > 190 K, but adsorbs at both ridge and terrace sites at *T* < 190 K. Therefore, at *T* > 190 K, a single GS is expected to appear for ridge sites after CH_3_OH dissociation, while at *T* < 190 K, double GSs for ridge and terrace sites are observed. Figure 4h compares the spectra obtained after CH_3_OH dissociation with samples prepared at 160 K and 220 K, respectively. Clearly, for the spectrum at  220 K, only one GS peak at ~1.20 eV can be detected; for the spectrum at 160 K, the peak is broadened to higher energies. The best fit can distinguish two peaks at about *E*_b_ ~ 1.20 eV and 1.75 eV (Fig. 4i), which could be assigned to the hydroxyl groups induced GSs at ridge and terrace sites, respectively. Such similarity strongly suggests that the excess electron doping by either O_V_ defects or hydroxyl groups can form a GS at *E*_b_ ~ 1.1–1.3 eV at the ridge and a GS at *E*_b_ ~ 1.6–1.8 eV at the terrace.

## Discussion

The above measurements unveil the surface electronic states in the intricated reconstructed surface of anatase-TiO_2_(001), which presents diverse O coordination environments. Using the multi-technique approach, we distinguish the bands of O_TERRACE_ and O_SIDE_ using the energy-momentum-resolved O *2p* spectra, the electronic states of O_BULK_, O_TOP-ADM_ and O_TOP-AOM_ using the O *2s* spectra, the defect electronic states at ridge and terrace sites through measuring the gap-state Ti *3d* spectra. These global parameters together establish the correlation of the surface electronic states with the (1 × 4) reconstructed structure of anatase-TiO_2_(001), and provide the deeper understanding of the mixed ADM-AOM configurations^36,37^. The revealed coordination (structure)-electronic (property) relationships could pave the way to understand the widely concerned surface catalysis and correlation phenomena of anatase-TiO_2_. For example, the main focus in photocatalytic water splitting on TiO_2_ is to understand the energy level alignment between the TiO_2_ VBs and the highest occupied molecular orbitals (HOMOs) of water at their interface^60^, which requires an insight into the intricate coordination environments and surface electronic structures. Distinguishing the surface VBs of O_TOP_, O_TERRACE_ and O_SIDE_ can directly locate and determine how the water HOMOs can hybridize with the TiO_2_ VBs with energy-momentum-site specificities; regarding the correlation phenomena, the anatase-TiO_2_ can provide a platform to tune the electron-phonon and electron-plasmon couplings through the excess charge by O_V_ defect^39,61^ and photo-excitation^62,63^, and can support an enhanced electron-phonon coupling interface of FeSe/anatase-TiO_2_ to induce high-*T*_c_ superconductivity^64^. Distinguishing the origins of the doped electrons from different GSs can provide prerequisites to tune the electron-boson couplings in the complicate many-body interactions. In summary, our results provide the comprehensive understanding of the complex coordination and the corresponding electronic structures of the prototypical anatase-TiO_2_(001), and beyond, may benefit to other TMO materials.

## Methods

### Sample preparation

The anatase-TiO_2_(001) thin films were epitaxially grown on 0.7 wt% Nb-doped SrTiO_3_(001) substrates by pulse laser deposition (PLD) method^39^. During deposition, The O_2_ pressure was kept at 1.5 × 10^−3 ^Pa and the substrate temperatures were kept at a 650 °C. To obtain high quality 1 × 4 reconstructed surface, the anatase-TiO_2_ films were grown slowly on SrTiO_3_ substrate with deposition rate of ~1 nm/h. The typical thickness of the thin films was 20–30 nm. The as-grown anatase-TiO_2_(001) samples were transferred between the PLD, STM and ARPES systems without exposure to air, via a portable ultrahigh vacuum transfer chamber with a battery powered ion pump to maintain a pressure better than 1 × 10^−9^ mbar.

### STM, XPS, and ARPES measurements

The STM measurements were conducted in a low temperature STM (Omicron LT, Matrix) at 80 K with a tungsten tip. High-resolved in situ STM and AFM experiments with a qPlus sensor were conducted at 5 K. The photoelectrons in XPS and ARPES measurements at 90 K were excited by the Mg Kα radiation (1253.6 eV) and the resonance He lamp (21.2 eV and 40.8 eV), respectively. The *hυ*-dependent XPS measurements were performed at the beam line 09U of Shanghai Synchrotron Radiation Facility (SSRF), with the photon energy varying from 39 to 610 eV with horizontally polarized light at 20 K. Hemispherical energy analyzer (VG Scienta, DA30L) was used for all measurements. Energy resolution of instrument was better than 5 meV and the angular resolution was better than 0.1°.

### DFT calculation

All the calculations are performed with the Vienna Ab-initio Simulation Package (VASP). Electron-nuclei interactions are described by the projector-augmented wave pseudopotentials^65^. The Perdew-Burke-Ernzerhof (PBE) exchange-correlation functions are employed in all calculation^66^. The long-range van der Vaals interactions are corrected with Grimme’s D2 method^67^. The Kohn-Sham wave functions are expanded in plane waves up to 500 eV. The structures are relaxed until the atomic forces are less than 0.01 eV/atom and total energies are converged to 10^−5 ^eV. A 15 Å thick area of vacuum in the z-direction and dipole correction are employed to avoid non-physical interaction between neighboring slabs.

#### Band calculation

The 3D BZs are sampled with a grid of 13 × 13 × 13 k-points according to the Monkhorst-Pack procedure for primitive cell and unit-cell of bulk anatase-TiO_2_, respectively^68^. The anatase-TiO_2_ (001)-(1 × 4) surface was modelled using a slab of four O-Ti-O trilayers based on ADM and AOM model, with the bottom O-Ti-O trilayer being fixed and 3 × 9 × 1 k-points are used for this surface. To assign the three new peaks in detail, we calculate the electronic structures using DFT with the hybrid HSE06 functional.

#### Surface energy and phase diagram calculation

Using the (4 × 4) supercell, we investigate different oxidized surfaces for ADM: AOM corresponding to ADM (not oxidation), 3:1 (25% oxidation), 1:1 (50% oxidation), 1:3 (75% oxidation) and AOM (all oxidation). The stability of these structures in different environments can be discussed by calculating the free energy correlation with the oxygen chemical potential. The chemical potentials must satisfy the following boundary conditions: (i) ${\mu }_{{{{{{\rm{O}}}}}}}\le \frac{1}{2}{E}_{{{{{{{\rm{O}}}}}}}_{2}}$; (ii) ${\mu }_{{{{{{\rm{Ti}}}}}}}\le {\mu }_{{{{{{\rm{Ti}}}}}}}^{{{{{{\rm{bulk}}}}}}}$ and (iii) ${\mu }_{{{{{{\rm{Ti}}}}}}}+2{\mu }_{{{{{{\rm{O}}}}}}}={E}_{{{{{{{\rm{TiO}}}}}}}_{2}}$, in which ${E}_{{{{{{{\rm{TiO}}}}}}}_{2}}$ is the internal energy of the bulk TiO_2_ per unit cell. The stability of surface energy can thus be written as:

1 $$\gamma=\frac{1}{N}({E}_{{{{{{\rm{tot}}}}}}}-{E}_{{{{{{\rm{ref}}}}}}}-{n}_{{{{{{\rm{Ti}}}}}}}{\mu }_{{{{{{\rm{Ti}}}}}}}-{\mu }_{{{{{{\rm{O}}}}}}}{n}_{{{{{{\rm{O}}}}}}})$$

According to the constraints of the chemical potential, the range of ${{{{{{\rm{\mu }}}}}}}_{{{{{{\rm{O}}}}}}}$ is set as ${\mu }_{{{{{{{\rm{O}}}}}}}_{2}}/2\ge {\mu }_{{{{{{\rm{O}}}}}}}\ge ({E}_{{{{{{{\rm{TiO}}}}}}}_{2}}-{\mu }_{{{{{{\rm{Ti}}}}}}})/2$. In equilibrium with O_2_ gas, ${\mu }_{{{{{{\rm{O}}}}}}}$ is expressed as:

2 $${\mu }_{{{{{{\rm{O}}}}}}}=\frac{1}{2}[{E}_{{{{{{{\rm{O}}}}}}}_{2}}+{\Delta H}_{{{{{{{\rm{O}}}}}}}_{2}}(T,\, {P}^{0})-{T\Delta S}_{{{{{{{\rm{O}}}}}}}_{2}}(T,\, {P}^{0})+{k}_{{{{{{\rm{B}}}}}}}T{{{{\mathrm{ln}}}}}(P/{P}^{0})]$$

where ${k}_{{{{{{\rm{B}}}}}}}$, ${P}^{0}$ and $P$ are the Boltzmann constant, standard atmospheric pressure, and oxygen partial pressure, respectively. ${E}_{{{{{{{\rm{O}}}}}}}_{2}}$ is the total energy of the O_2_ obtained from a spin-polarized calculation. ${\Delta H}_{{{{{{{\rm{O}}}}}}}_{2}}(T,{P}^{0})$ and ${T\Delta S}_{{{{{{{\rm{O}}}}}}}_{2}}(T,{P}^{0})$ are taken from a thermodynamic database^69^. The last term is the contribution coming from the partial pressure of oxygen.

#### O_V_ defects calculation

The geometry optimizations and electronic states of O_V_ defects at the ridge and terrace sites are simulated in 4 × 3 supercell, which supplies enough room to distort the lattice for electron. We perform the spin-polarized DFT calculation using the PBE method to calculate the configurations and formation energies of possible surface O_V_ configurations. Due to the self-interaction error^70^, the orbitals are too delocalized in DFT calculation to locate an electron to an individual Ti atom. To overcome this problem, we perform the spin-polarized DFT calculation using the PBE + U method with U = 3.9 eV^40^ to calculate pDOS and electronic charge distribution. After geometry optimization, we use velocity rescaling to bring the temperature of the system to 300 K. An 8 ps microcanonical ab initio molecular dynamics (AIMD) trajectory is generated.

### Supplementary information

Supplementary Information

Peer Review File

### Source data

Source Data

## Data Availability

All data needed to support the conclusions in the study are available within the article and/or the supplementary files. Data underlying Figs. 1–4 and Supplementary Figs. 1–14 are provided in the source data file with this paper. Source data are provided in this paper.
