# Supplementary material for: Unveiling diverse coordination-defined electronic structures of reconstructed anatase TiO2(001)-(1 × 4) surface
Source: Nat Commun. 2024 Mar 14;15:2326. doi: 10.1038/s41467-024-46570-8 (PMC10940315; doi:10.1038/s41467-024-46570-8)
Supplement: Supplementary file 1 — Supplementary Information [file 41467_2024_46570_MOESM1_ESM.pdf]

Supplementary Information

Unveiling diverse coordination-defined electronic structures of reconstructed anatase TiO_2(001)-(1×4) surface

Xiaochuan Ma[1,2,5], Yongliang Shi[1,5], Zhengwang Cheng[3,5], Xiaofeng Liu[4], Jianyi Liu[1], Ziyang Guo[1,2], Xuefeng Cui[1,2], Xia Sun[1,2], Jin Zhao[1,2], Shijing Tan[1,2] ✉ and Bing Wang[1,2] ✉

[1]Hefei National Research Center for Physical Sciences at the Microscale and New Cornerstone Science Laboratory, University of Science and Technology of China, Hefei, Anhui 230026, China

[2]Hefei National Laboratory, University of Science and Technology of China, Hefei 230088, China

[3]School of Science and Hubei Engineering Technology Research Center of Energy Photoelectric Device and System, Hubei University of Technology, Wuhan 430068, China

[4]School of Physics, Hefei University of Technology, Hefei, Anhui 230009, China

[5]These authors contributed equally: Xiaochuan Ma, Yongliang Shi, Zhengwang Cheng.

✉ e-mail: tansj@ustc.edu.cn; bwang@ustc.edu.cn

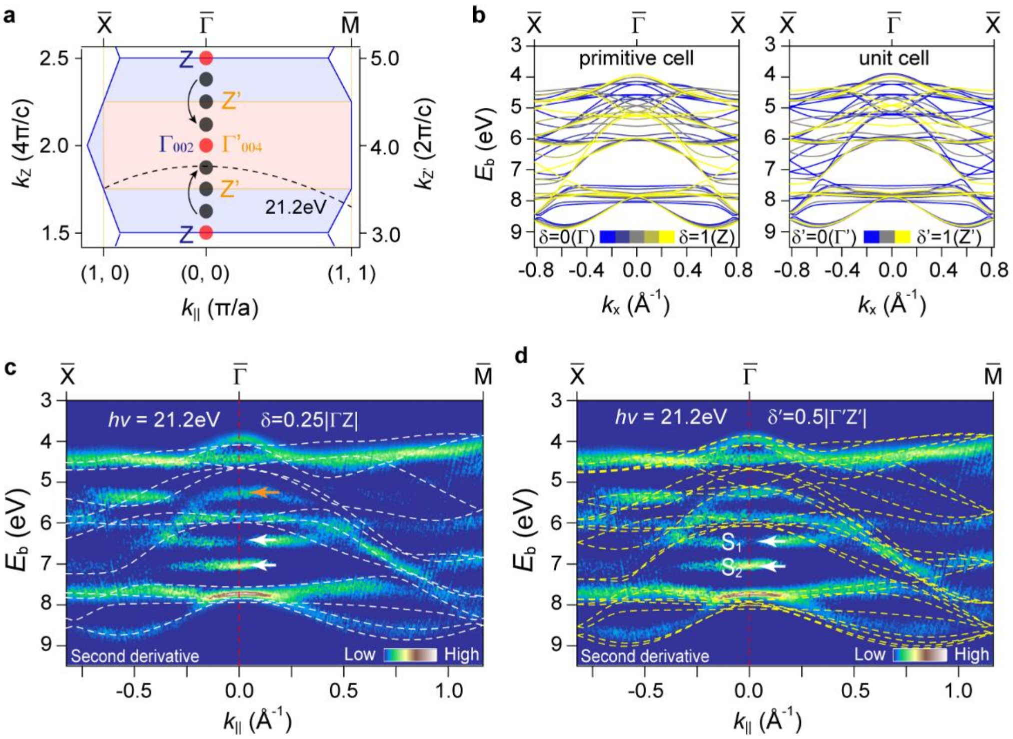

Supplementary Figure 1. Calculated and measured VBs of anatase-TiO_2 (001). a The $k_{||}k_z$ BZ map along $\bar{\Gamma}-\bar{X}$ and $\bar{\Gamma}-\bar{M}$ directions, corresponding to the [100] and [110] directions of the anatase-TiO_2(001) surface, respectively. The black arc traces the trajectory of $k_{||}k_z$ constant energy (CE) map at $E_b \sim 6$ eV (center level of VBs) measured by $h\upsilon = 21.2$ eV. The position of black arc at $\bar{\Gamma}$ point is around $k_z = \delta|\Gamma Z| + \Gamma_{002}$ with $\delta \sim 0.25$ for BZ of primitive cell (blue, left axis) and $k_z' = \delta'|\Gamma'Z'| + \Gamma_{004}'$ with $\delta' \sim 0.5$ for BZ of unit cell (orange, right axis), respectively. The curved arrows indicate the band folding along the k_z direction in the unit cell. **b** The calculated bulk VBs of primitive cell (left panel) and unit cell structures (right panel) with different k_z, respectively. Since the unit cell has a volume double of the primitive cell, the total number of the calculated VBs of unit cell (24 bands) is twice of the number of primitive cell (12 bands) due to the BZ folding. **c,d** The second derivative spectrum, overlaid with the calculated bulk VBs of the primitive cell at $\delta \sim 0.25$ (white dashed lines) and of the unit cell at $\delta' \sim 0.5$ (yellow dashed lines), respectively.

The calculated bulk VBs with unit cell at $\delta' \sim 0.5$ contain double bands, which show better overlap with the measured results for the band at $E_b \sim 5.2$ eV (yellow arrow), confirming their origin from the band folding along k_z direction. The other two bands of S_1 at $E_b \sim 6.5$ and S_2 at $E_b \sim 7.1$ eV (white arrows) still cannot be assigned to any calculated bulk VBs, indicating their surface origins.

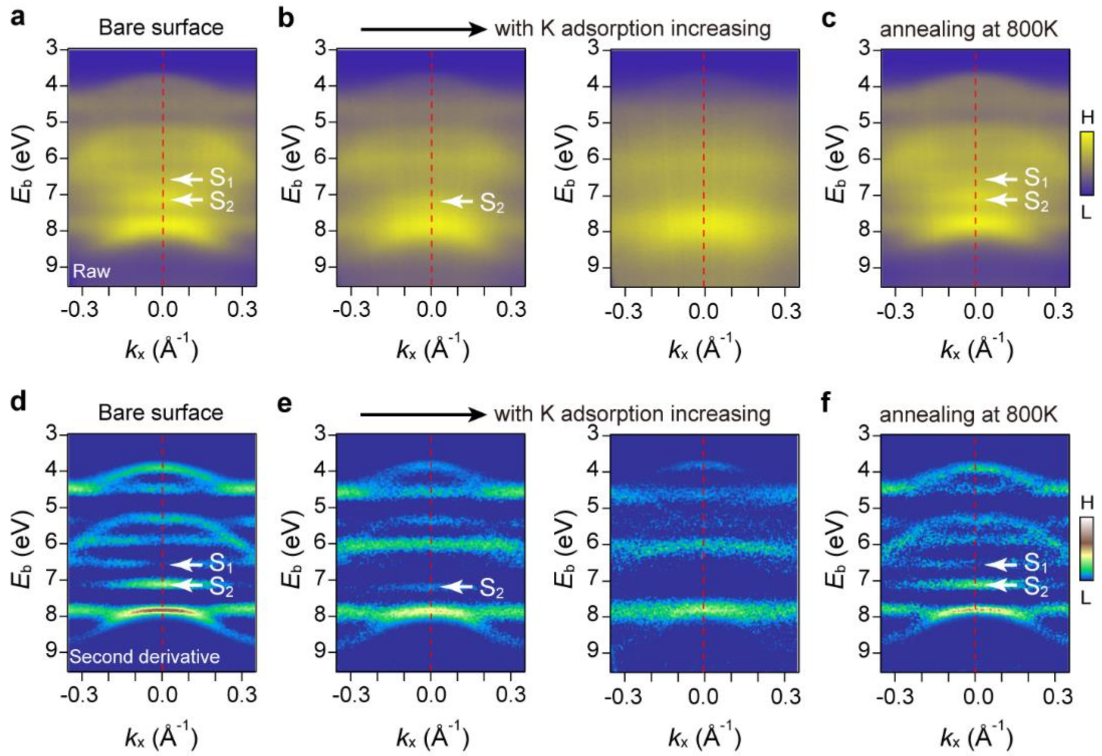

Supplementary Figure 2. Demonstration of S1 and S2 as surface bands by potassium (K) adsorption. a-c The raw ARPES cuts of bare anatase-TiO_2(001) surface (**a**), after K adsorption with increasing coverage (**b**), and after annealing at 800 K (**c**). **d-f** The corresponding second derivative maps of (**a**-**c**).

Introducing K atoms to surface is a conventional method that can not only introduce electron doping, but also eliminate the surface states. It is observed the S_1 and S_2 bands are gradually quenched after the adsorption of K atoms, while the other bands remain almost unchanged. After annealing to 800 K, the S_1 and S_2 are recovered, indicating the desorption of K atoms from surface.

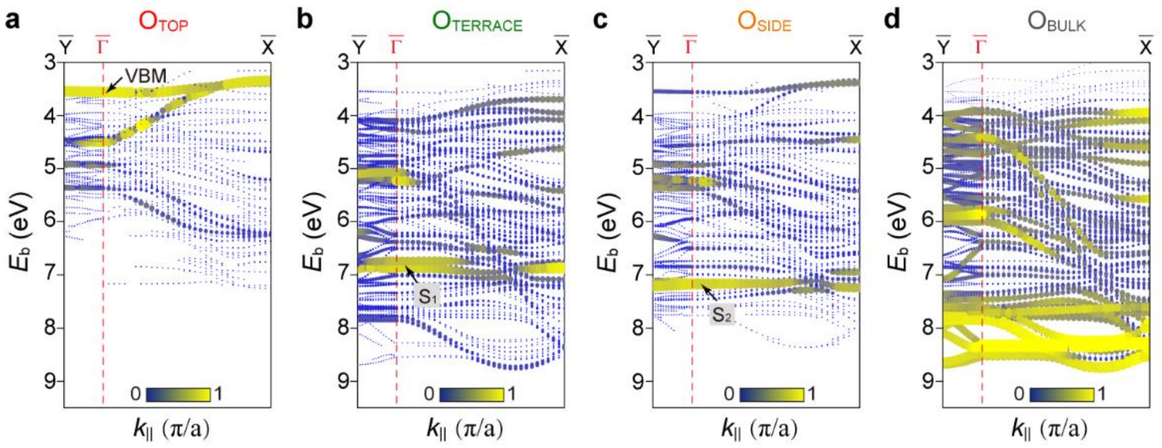

Supplementary Figure 3. Calculated VB dispersion according to the weightings of OTOP, OTERRACE, OSIDE and OBULK based on ADM model. The weightings of surface O atoms (**a**-**c**) show local distribution relative to the that of bulk O atoms (**d**). The weightings of S_1 and S_2 indicate near flat character in the vicinity of $\bar{\Gamma}$ point.

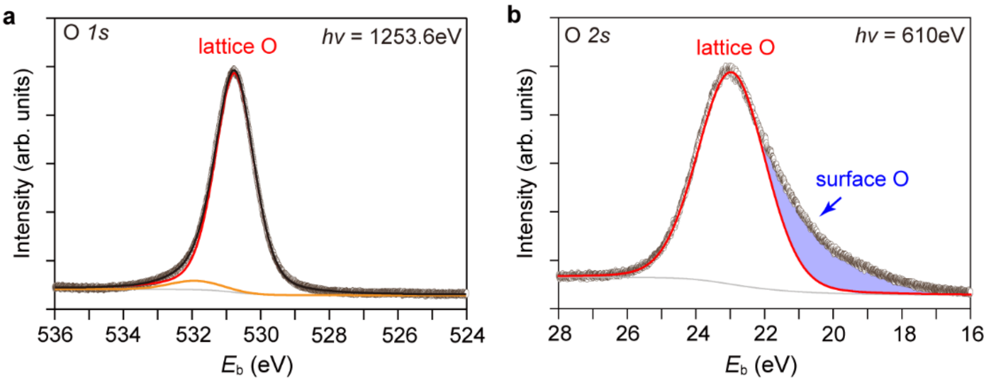

Supplementary Figure 4. Comparing the O *1s* and O *2s* spectra of anatase-TiO_2(001) surface. **a,b** Typical O *1s* and *2s* spectra with excited by $h\upsilon$ = 1253.6 and $h\upsilon$ = 610 eV, respectively. With the best XPS fitting, the O *1s* spectrum presents a dominant peak at E_b ~ 530.6 eV (red) and a weak high E_b tail (brown, +1.1 eV). By contrast, the O *2s* semi-core-level spectrum presents a broad side peak spreading from E_b ~ 17 to 22 eV (blue shaded), giving an opportunity to analyze the different electronic states from different surface O coordination.

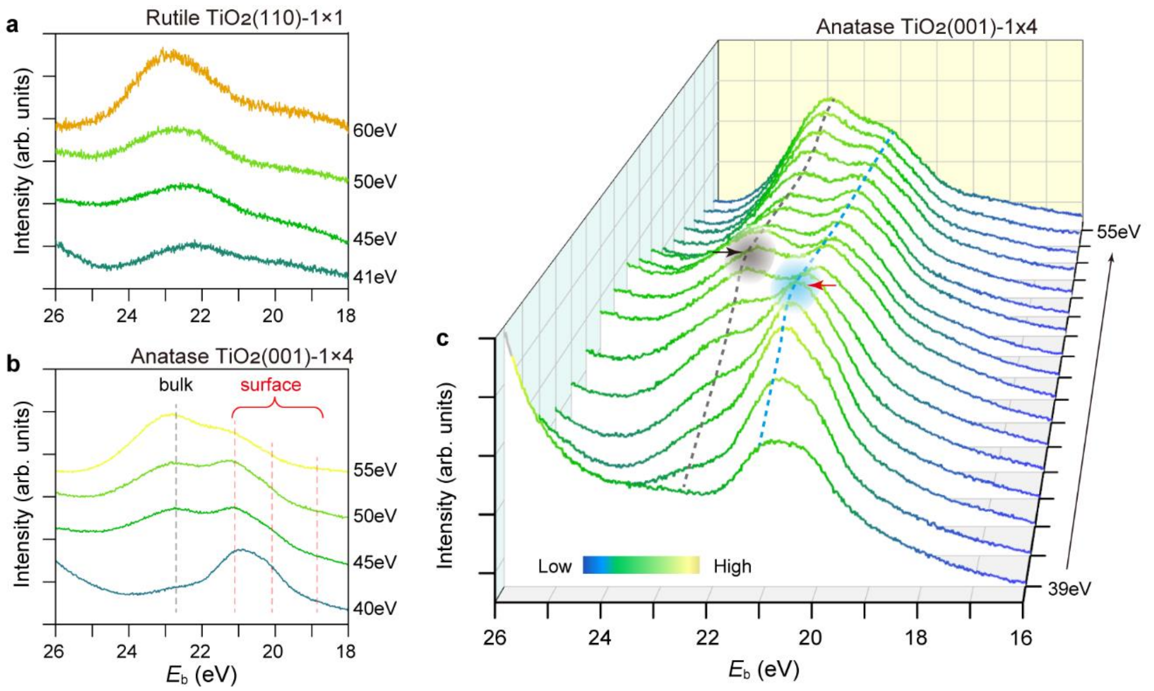

Supplementary Figure 5. O *2s* spectra and resonant photoemission process. a The measured O 2s XPS of rutile-TiO_2(110)-(1×1) excited by *hυ* = 41, 45, 50 and 60 eV, respectively. **b** The corresponding data for anatase-TiO_2(001)-(1×4) excited by *hυ* = 40, 45, 50 and 55 eV, respectively. Anatase-TiO_2(001)-(1×4) surface shows multiple peaks, while rutile-TiO_2(110)-(1×1) surface is featureless with a broad peak. **c** Evolution of O 2s XPS by varying the photon energy from 39 eV to 55 eV in 1 eV/step (the raw data is from the same set of experiments of Fig. S4c in ref. 39. Here, we analyze the resonant feature at 43-46 eV). The strength of bulk increases with the increase of the excitation energy (gray line). But, resonant photoemission process occurs at around *hυ* ~ 43 eV for surface level (red arrow) and *hυ* ~ 46 eV for bulk level (black arrow). Such a resonant photoemission process could enhance the photoemission intensities of these surface semi-core levels, and make the peaks more distinguishable in the O *2s* spectra. All measurements were performed at 20 K.

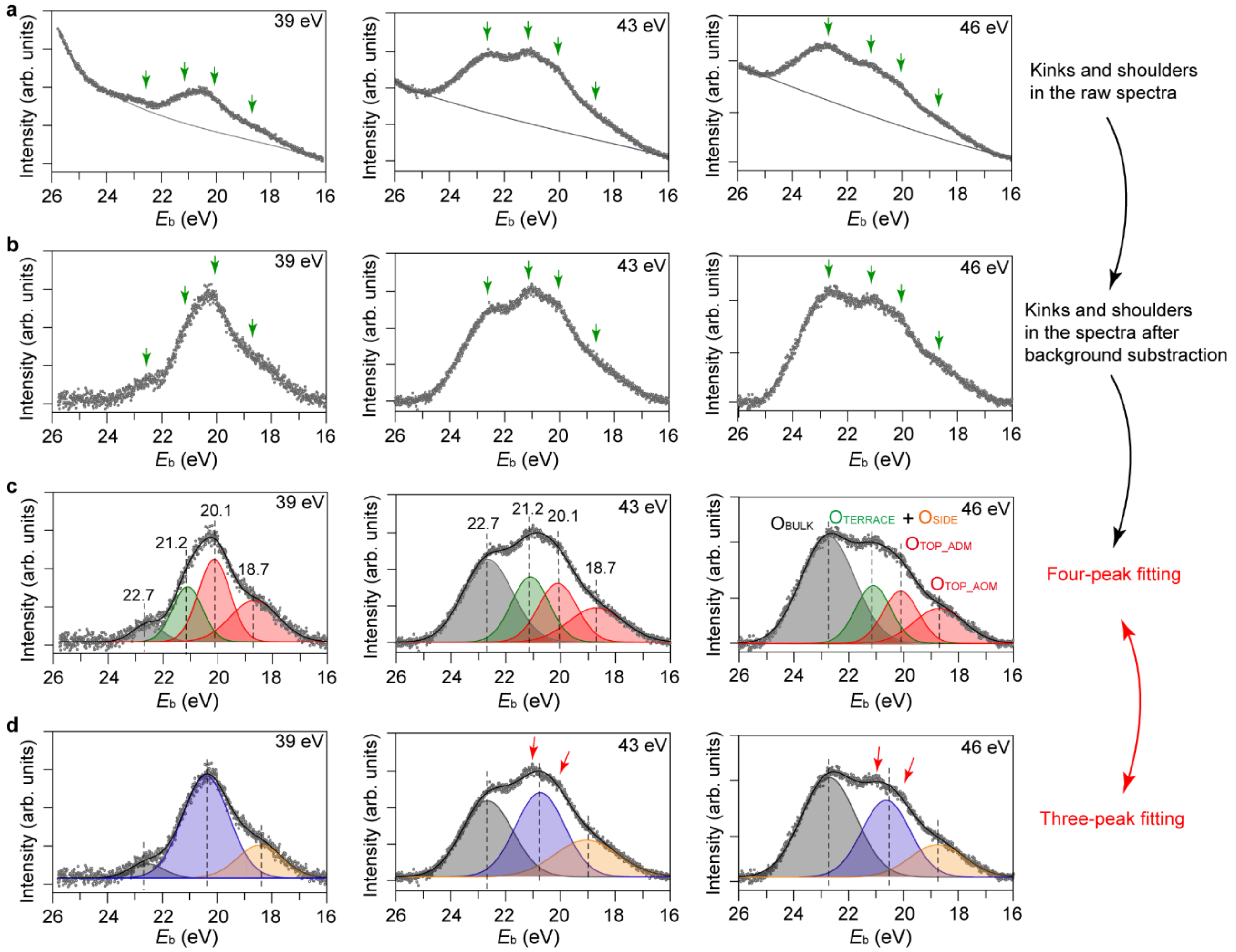

Supplementary Figure 6. Peak fitting of O *2s* spectra. a The measured O *2s* XPS of anatase-TiO_2(001)-(1×4) excited by *hυ* = 39, 43 and 46 eV, respectively. The backgrounds (BGs) to be subtracted are shown with grey lines. **b** The corresponding spectra after BG subtraction. The green arrows point to possible peaks. Peak fitting of O *2s* spectra for four-peak fit (**c**) and three-peak fit (**d**), respectively. (**c**) is same as Fig. 3b-d in the main text.

All the spectra in (**c**) can be decomposed into four peaks by the best XPS fitting. In a series of spectra with different excitation *hυ*, the corresponding peaks should keep at the same energy, but change in intensity, as labeled by the dashed lines. We tried a three-peak fit for the same spectra in (**d**). It can be found that the middle peak (blue shade) cannot well reproduce the two kinks at 21.2 and 20.1 eV (red arrows), and the energies of middle (blue shade) and right (brown shade) peaks are changing for different *hυ*. Thus, the three-peak fit is apparently worse than the four-peak fit.

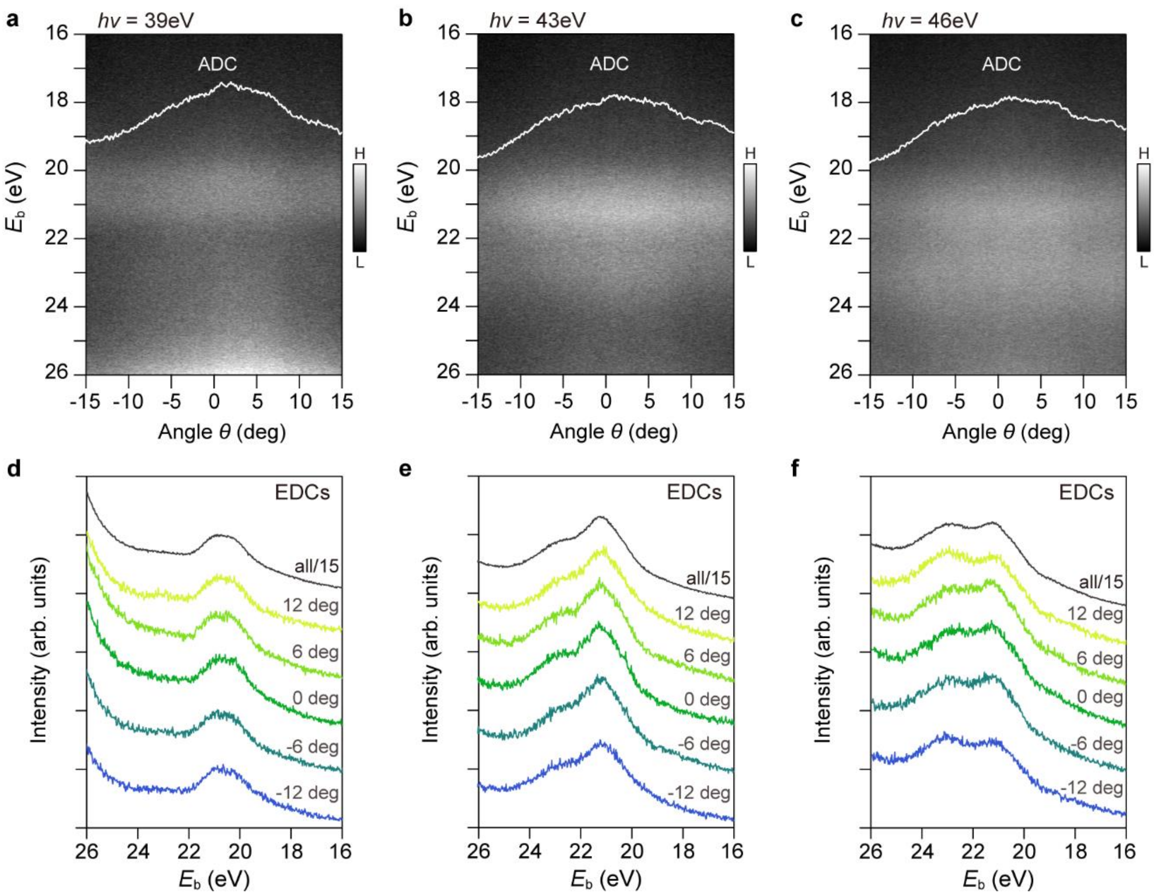

Supplementary Figure 7. Angle - and energy-dependent O *2s* spectra. a-c Three selected E_b-θ cuts with $h\upsilon$ = 39, 43 and 48 eV excitation. The angle distribution curves (ADCs) are marked by white lines (signal integration within the E_b range of 18-24 eV). **d-f** The corresponding energy resolution curves (EDCs) at different scattering angles (in each curve, the signals were integrated within ±1° at the labeled angle), in comparison with the normalized total signals (integrated signals with the whole range of ±15°). The EDCs show almost consistent spectral line profiles at different scattering angles, indicating the angle-independent O *2s* spectra.

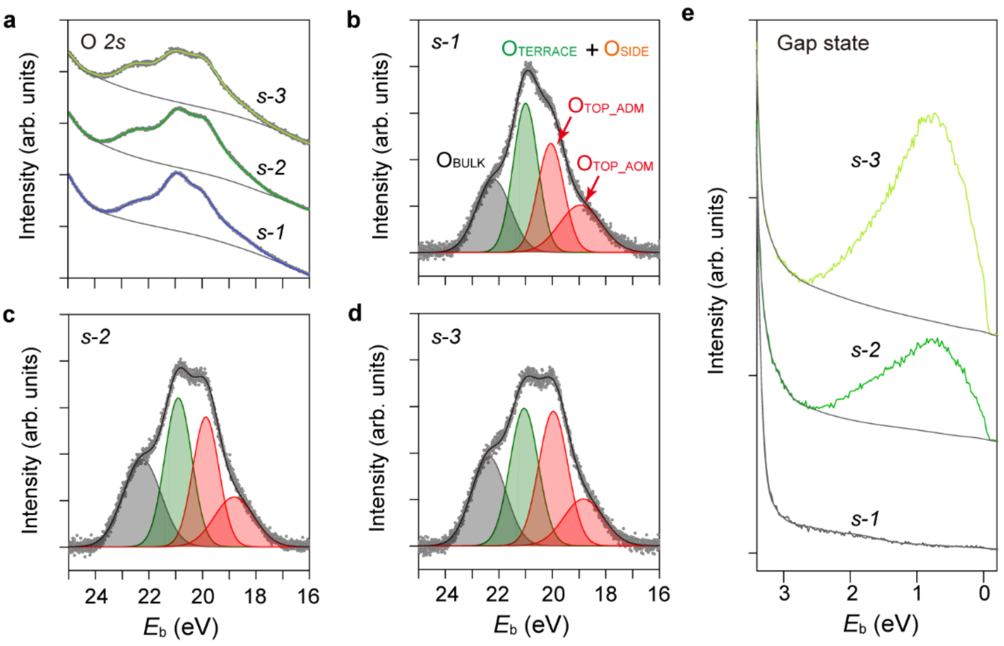

Supplementary Figure 8. Change the ADM:AOM ratio by slight sample reduction. a The raw O *2s* semi-core-level spectra of the anatase-TiO_2(001) surface with increased reduction excited by the resonance He 2α lamp (*hυ* = 40.8 eV). The sample reduction from *s-1* (as-grown) to *s-3* is produced by increasing Ar^+ sputtering (each cycle 2 min) and annealing at 650 ºC. The gray lines indicate the BG. **b-d** Peak fitting of O *2s* spectra with BG subtracted for *s-1* to *s-3* samples. The gray, green and red shades are defined in (**b**). The change of peak areas of the ADM and AOM components are plotted as a function of increasing reduction in Figure 3f in the main text. The ratio can be calculated via $I_{O_{TOP}}/(I_{O_{SURFACE}} + I_{O_{BULK}})$ for ADM and AOM ridges, where $I_{O_{TOP}}$, $I_{O_{SURFACE}}$ and $I_{O_{BULK}}$ are the area of O_{TOP}, surface O and bulk O contributed area, respectively. **e** The Ti *3d* in-gap state intensity of the samples *s-1* to *s-3* with increasing reduction.

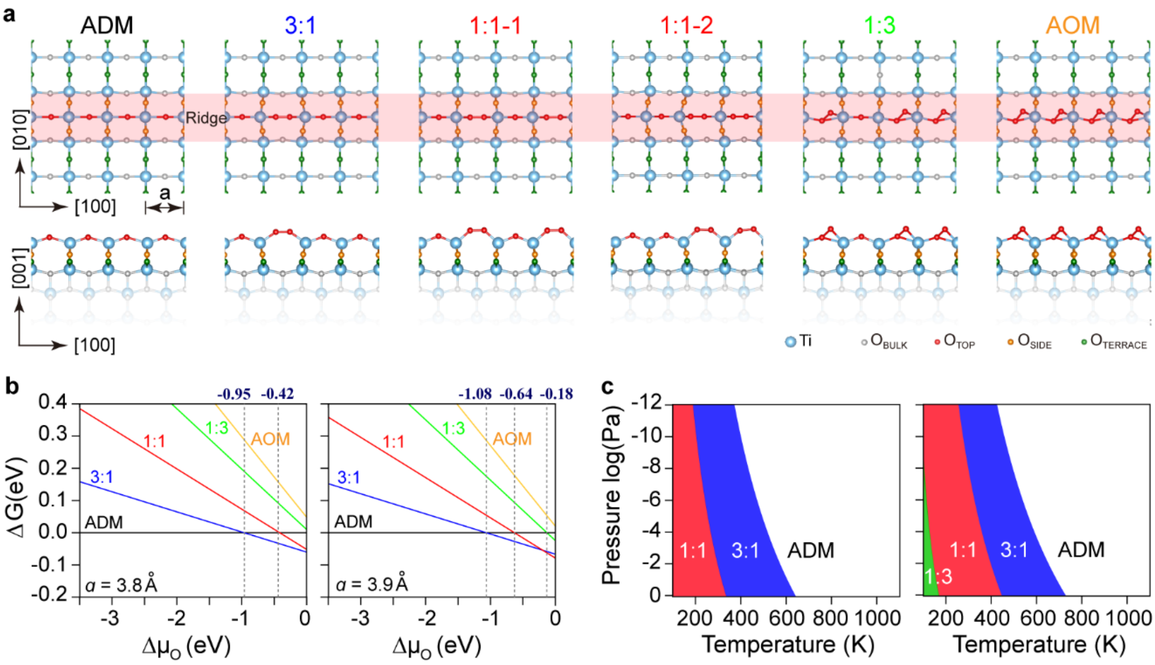

Supplementary Figure 9. The stabilities and phase diagrams of the mixed ADM-AOM structures. **a** The optimized structures of pure ADM, 3:1 (the numbers denote the ADM:AOM ratio); 1:1, 1:3 and pure AOM. The surface O atoms of O_{TOP}, O_{SIDE} and $O_{TERRACE}$ are colored by red, brown, and green, respectively. Although the two setups marked as 1:1-1 and 1:1-2 have different structures, they have nearly identical surface energies (difference within ~5 meV). **b** The calculated surface energies of the five structures in (**a**), plotted as a function of oxygen chemical potential $\Delta\mu_O$ for two different lattice constants of a = 3.8 Å (left panel) and a = 3.9 Å (right panel), respectively. A larger lattice constant a = 3.9 Å is used due to anatase-TiO_2(001) thin films epitaxially grown on 0.7 wt% Nb-doped $SrTiO_3$(001) substrates. **c** The corresponding phase diagrams with oxygen pressure and annealing temperature, calculated on the basis of the lattice constants of a = 3.8 Å (left panel) and a = 3.9 Å (right panel), respectively.

It can be seen that the ADM dominates when the annealing temperature is high and the oxygen pressure is low. When the annealing temperature is reduced and the oxygen pressure is increased, the mixed ADM-AOM structures appear and become stable. The results indicate that the mixed ADM-AOM structure is reasonable in sample growth.

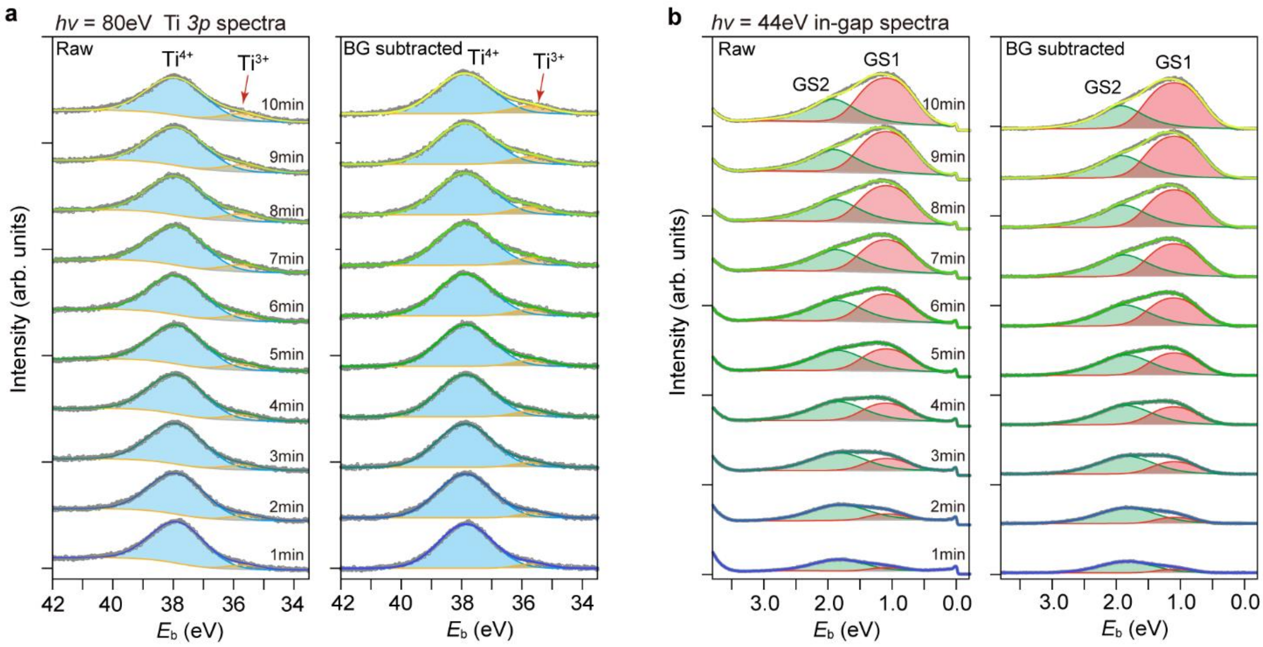

Supplementary Figure 10. Analysis of the Ti *3p* and Ti *3d* spectra with increasing oxygen vacancies under light irradiation on anatase-TiO_2(001)-(1×4) surface. a,b Data fitting of the raw (left panel) and BG subtracted (right panel) Ti *3p* and in-gap state spectra, respectively.

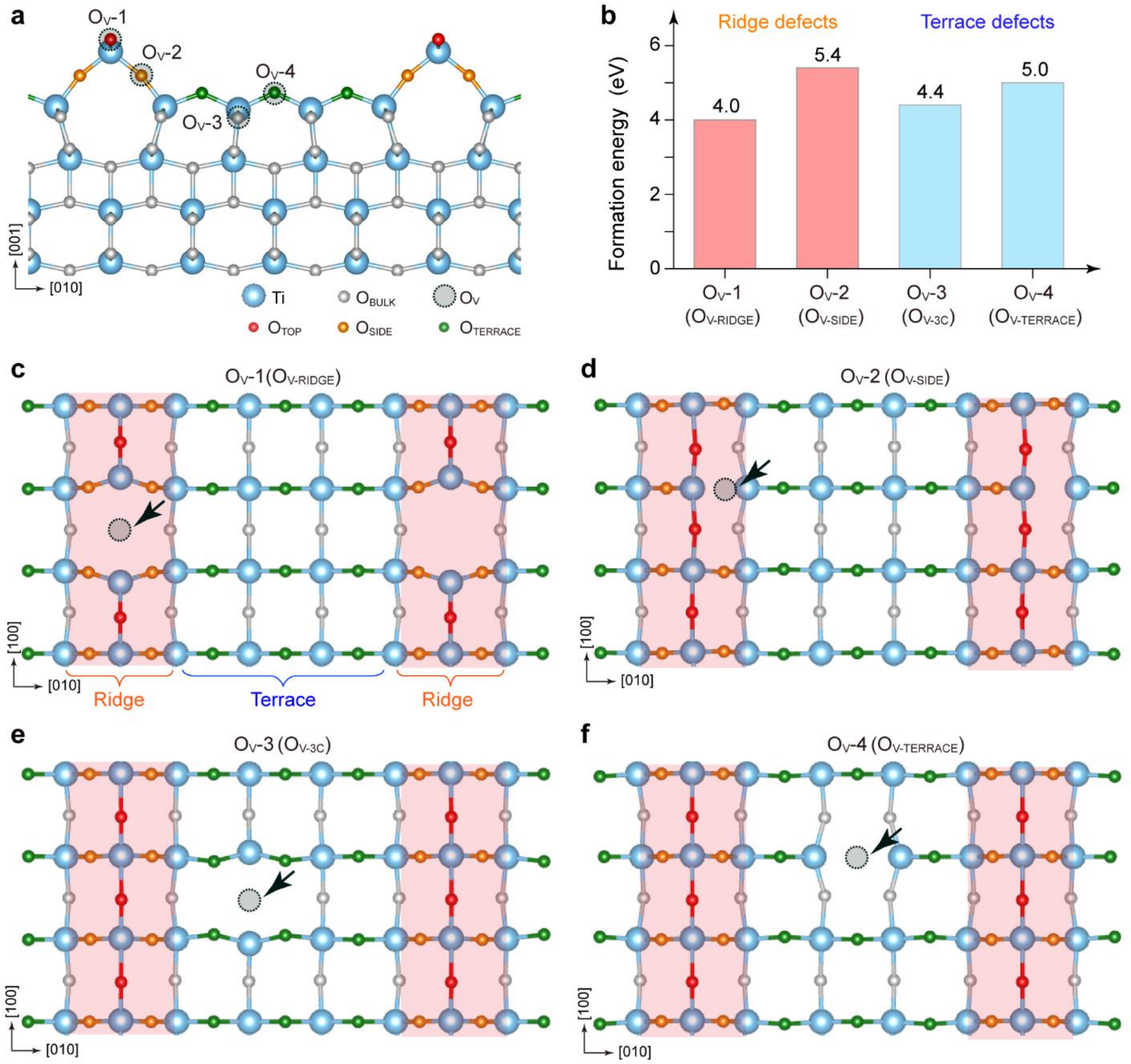

Supplementary Figure 11. Formation energies of possible surface O_V configurations. **a** Side view of the (1 × 4) reconstructed anatase TiO_2(001) slab model. All possible surface O_V configurations are represented with circles. The atoms of O_{TOP}, O_{SIDE}, $O_{TERRACE}$ and O_{BULK} are colored differently. **b** O_V formation energies (eV) at different surface sites. The O_V-1 ($O_{V\text{-}RIDGE}$) is the most likely to appear at the ridge sites, and the O_V-3 ($O_{V\text{-}3C}$) and O_V-4 ($O_{V\text{-}TERRACE}$) with energy difference of 0.6 eV may co-exist at the terrace sites. **c-f** Relevant O_V defect configurations for O_V-1 ($O_{V\text{-}RIDGE}$), O_V-2 ($O_{V\text{-}SIDE}$), O_V-3 ($O_{V\text{-}3C}$) and O_V-4 ($O_{V\text{-}TERRACE}$), respectively. O_V sites are marked by black arrows.

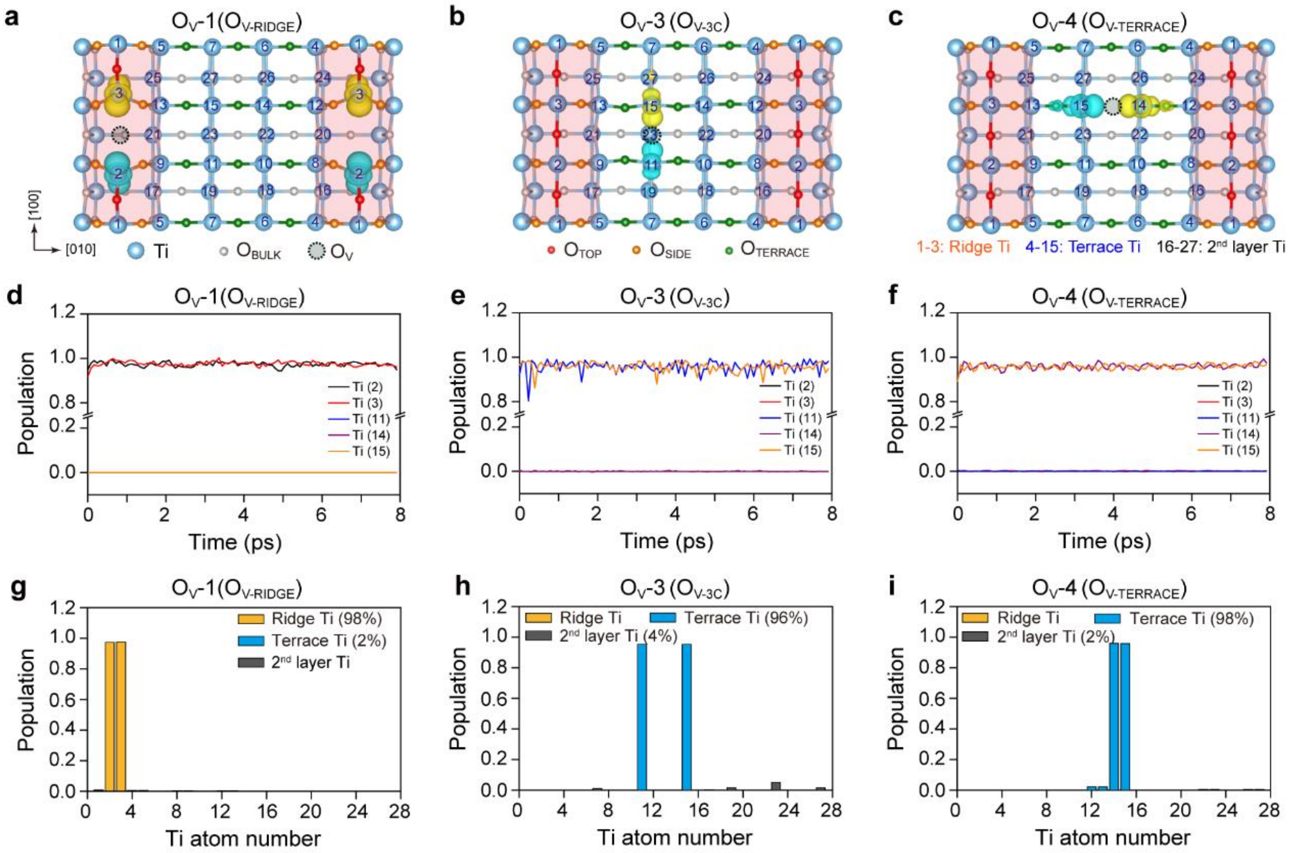

Supplementary Figure 12. The dynamical behavior of excess charge in three kinds of defective structures of $O_{V\text{-RIDGE}}$, $O_{V\text{-3C}}$ and $O_{V\text{-TERRACE}}$. a-c The initial charge distributions at $t = 0$ with the labeled Ti atom in three O_V defect configurations for $O_{V\text{-RIDGE}}$, $O_{V\text{-3C}}$ and $O_{V\text{-TERRACE}}$, respectively. **d-f** Dynamics of the fractional occupation of particular Ti 3*d* orbitals during a time fragment at $T = 300$ K; At $t = 0$ the charge is localized in the two nearest Ti atoms; populations of about 1 and 0 correspond to Ti^{3+} and Ti^{4+} charge states, respectively. **g-i** Distribution function of the average population of available Ti atoms by the two excess electrons obtained from the full simulation (**d**-**f**). The results show the excess charge on anatase-TiO_2(001)-(1×4) is highly localized, and eventually settles down to the two nearest Ti atoms (> 95%).

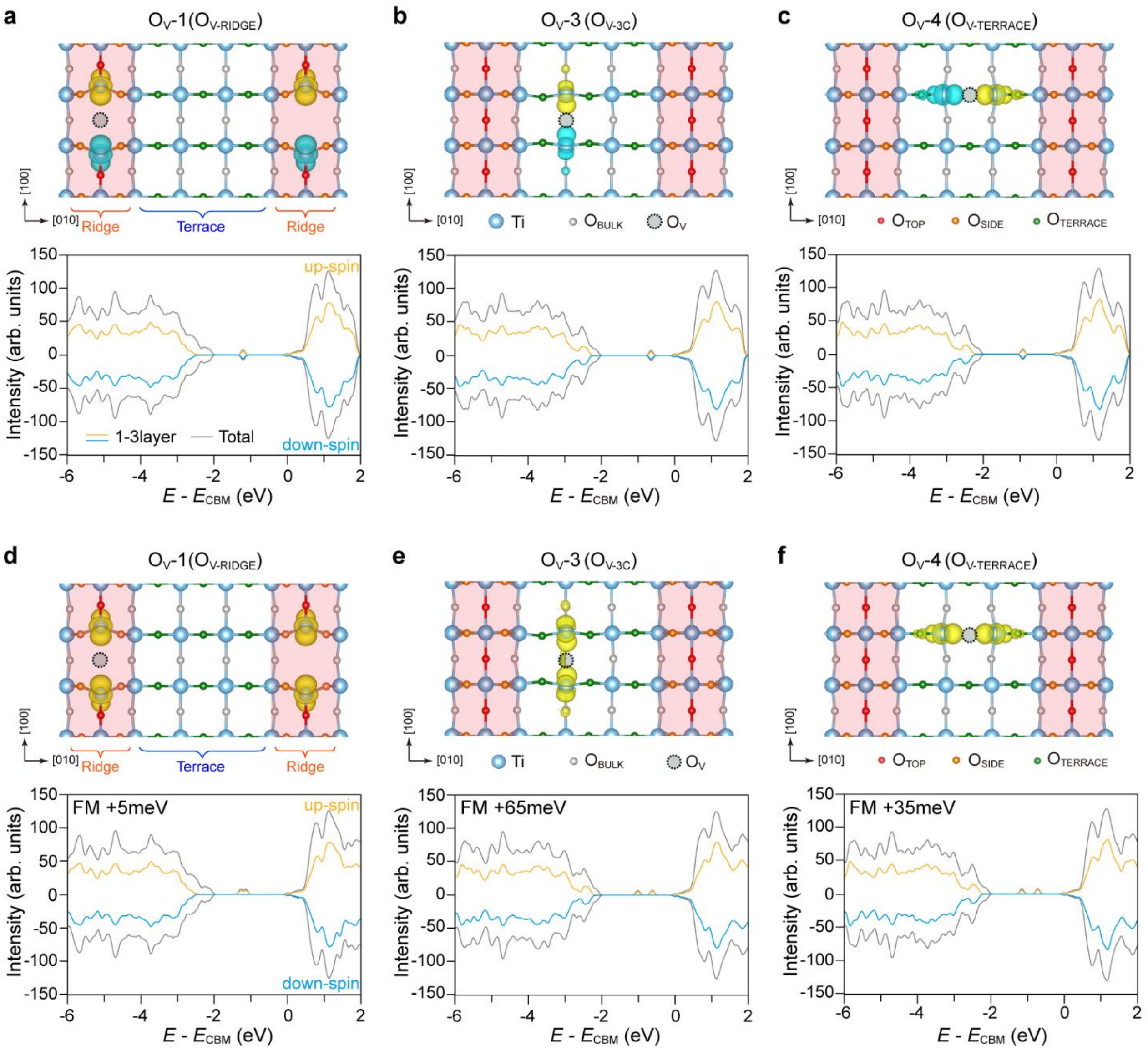

Supplementary Figure 13. The calculated GSs of different O_V sites with antiferromagnetic and ferromagnetic (FM) states. Calculated charge density contours of excess electrons and DOSs of total (gray) and 1-3 layer (yellow: spin up, blue: spin down) based on ADM model with an O_V-1 ($O_{V\text{-RIDGE}}$) at ridge (**a,d**), an O_V-3 ($O_{V\text{-3C}}$) at terrace (**b,e**) and an O_V-4 ($O_{V\text{-TERRACE}}$) at terrace (**c,f**), respectively. The antiferromagnetic state (**a-c**) has a lower formation energy than the ferromagnetic (FM) state (**d-f**). DOS of 1-3 layers is extracted to eliminate the effects of (1×1) surface at the last layer, the calculated bandgap is estimated to ~ 2.5 eV. Charge density contours of the excess electron states are induced by O_V defects. The excess electrons are mainly distributed in adjacent Ti atoms, reducing the Ti^{4+} to Ti^{3+}, implying the formation of a small electron polaron.

From the pDOS with either antiferromagnetic or FM states, the energies of different O_V defects are not separated clearly, making the assignment difficult.

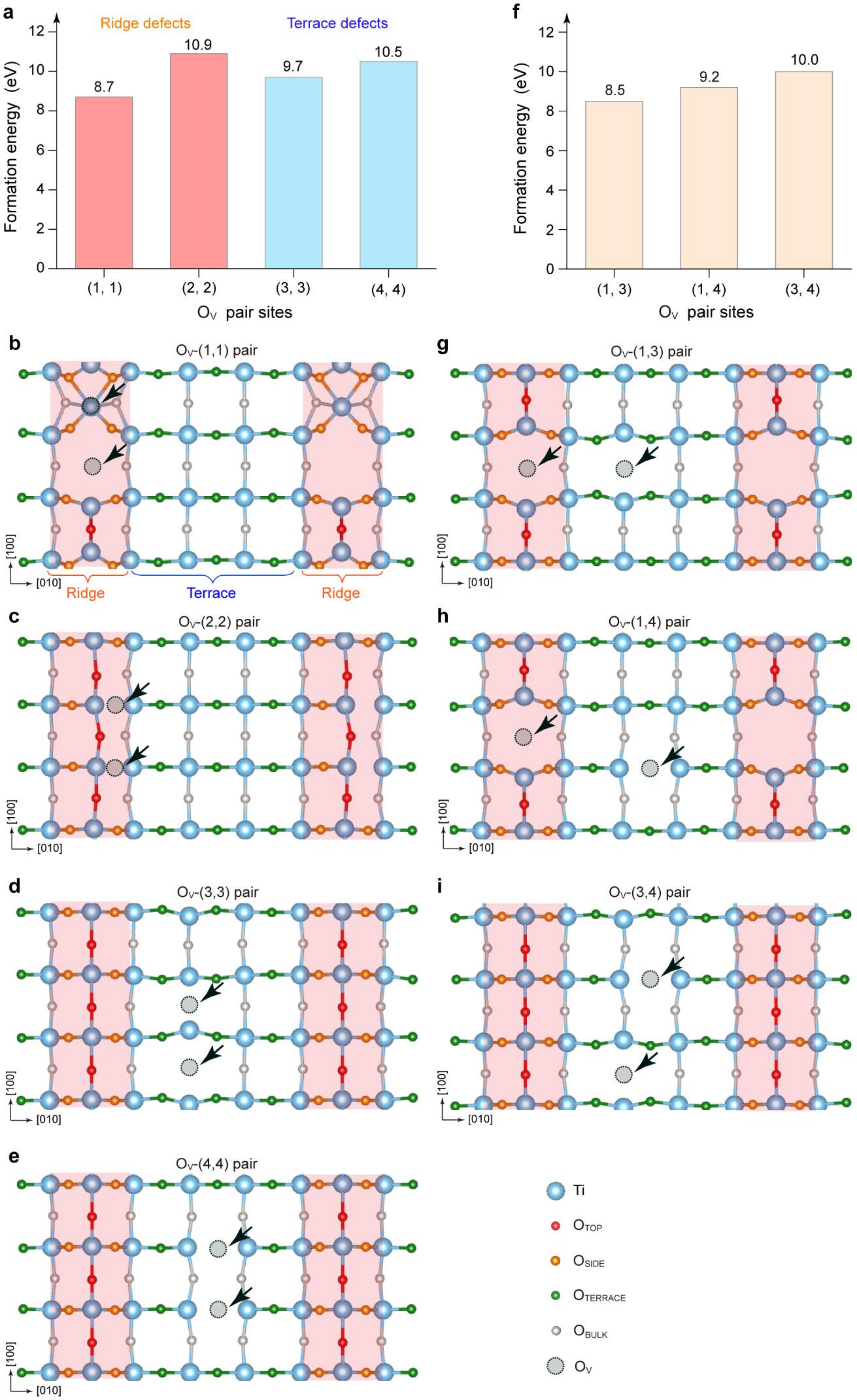

a
Ridge defects
Terrace defects
Formation energy (eV)
8.7
10.9
9.7
10.5
(1, 1)
(2, 2)
(3, 3)
(4, 4)
O_V pair sites
f
Formation energy (eV)
8.5
9.2
10.0
(1, 3)
(1, 4)
(3, 4)
O_V pair sites
b
O_V-(1,1) pair
Ridge
Terrace
Ridge
[100]
[010]
c
O_V-(2,2) pair
d
O_V-(3,3) pair
e
O_V-(4,4) pair
g
O_V-(1,3) pair
h
O_V-(1,4) pair
i
O_V-(3,4) pair
Ti
O_TOP
O_SIDE
O_TERRACE
O_BULK
O_V

Supplementary Figure 14. Formation energies of multiple O_V defects. Formation energies of same (**a**) and different (**f**) kinds of O_V pairs. **b-e** Relevant same kind of O_V pair configurations for O_V-(1,1), O_V-(2,2), O_V-(3,3) and O_V-(4,4) pairs, respectively. **g-i** Relevant different kinds of O_V pair configurations for O_V-(1,3), O_V-(1,4) and O_V-(3,4) pairs, respectively.

For different kinds of O_V pairs, the O_V-(1,3) and O_V-(1,4) pairs have the lower formation energy. For same kind of O_V pairs, the O_V-(1,1) pair at ridge sites has the lowest formation energy of 8.7 eV. Experimentally, the intensity of GS1 can continue to increase while that of GS2 quickly achieve saturation (Fig. 4d in the main text), suggesting that GS1 related defects can be reasonably assigned to ridge sites.
